# Surface Physical and Chemical Modification of Pure Iron by Using Atmospheric Pressure Plasma Treatment

**DOI:** 10.3390/ma13214775

**Published:** 2020-10-26

**Authors:** Jinxing Kong, Dongxing Du, Aisheng Song, Fan Zhang, Wen Huang

**Affiliations:** 1 Institute of Machinery Manufacturing Technology, China Academy of Engineering Physics, Mianyang 621999, China; kongjx0058@yinhe596.cn (J.K.); 18842814603@163.com (F.Z.); 2 State Key Laboratory of Tribology, Tsinghua University, Beijing 100084, China; sas17@mails.tsinghua.edu.cn

**Keywords:** atmospheric pressure plasma treatment, pure iron, surface wettability, tensile property, nanomechanics, molecular dynamics simulation

## Abstract

To investigate the mechanism of surface modification of pure iron by atmospheric pressure plasma treatment (APPT), the surface wettability of pure iron was characterized by using a contact-angle measuring instrument, and the mechanical properties of pure iron were measured by a tensile testing machine and nanoindentation instrument. Molecular dynamics simulations were used to explain the modification mechanism of the surface wettability and the mechanical behavior of pure iron by APPT. The experimental results show that pure iron treated by APPT is superhydrophilic, with reduced tensile strength and surface hardness. This result agrees with the molecular dynamics simulation, which shows that the pure iron material hydrophilicity improved after APPT. The behavior was attributed to the formation of hydrogen bonds on the surface of the pure iron after APPT. The surface binding energy of the pure iron material increased between the water molecule and the residual N atom that was induced by APPT. The N atom that was introduced by the APPT led to Fe bond fracture, and the N atom reduced the Fe bond strength, which resulted in a reduction of material yield strength and microhardness.

## 1. Introduction

Pure iron has an excellent plasticity, impact toughness and electromagnetic properties, and is used widely in biology, electronics, aerospace and other fields [1,2,3]. However, during the cutting of pure iron, large deformations and work-hardening phenomena occur. A built-up edge forms on the front cutter face, which yields a large cutting force and cutting temperature. The chips are continuous and strong, and the tool is worn rapidly, which makes it difficult to guarantee the surface quality and dimensional accuracy of the workpiece. Therefore, pure iron is a typical material that is difficult to process [4,5,6]. To resolve these problems, during the cutting of pure iron, predominant technical methods were investigated, including dry-ice technology, minimum-quantity-lubrication (MQL) technology, and water-jet technology. These technical means reduce the cutting area temperature, improve the cutting performance and extend the tool service life [7,8,9,10,11,12]. Dry ice is used to reduce tool wear when cutting pure iron. However, liquid carbon-dioxide cooling and the transmission equipment required for this method have strict requirements in terms of sealing and gas pressure, rendering the device complex [7,8,9]. Kong et al. [10,11,12] studied the notch wear mechanism of cemented carbide tools when wet cooling, and MQL was used to assist in the cutting of pure iron materials. Therefore, wet cooling increases tool notch wear, whereas MQL can reduce the tool notch wear, but the degree of reduction is limited.

Atmospheric pressure plasma treatment (APPT) is conducive to the auxiliary cutting of some difficult-to-cut materials [13,14,15,16,17]. The atmospheric pressure plasma is rich in active particles and has a low temperature, which improves the metal surface wettability rapidly and improves the cooling and lubricating environment in the cutting area. The cutting process is environmentally friendly, so it has a broad application prospect in the auxiliary precision machining of pure iron and other materials. Katahira et al. [13] studied the micromilling of SiC with polycrystalline diamond tools. The surface roughness (*Ra*) of the workpiece could be reduced from 3.22 nm to 0.73 nm by atmospheric pressure plasma technology, and the polycrystalline diamond tool life could be improved. Bastawros et al. [14] applied a He-H_2_O mixed atmospheric pressure plasma jet to assist with sapphire polishing, and found that the material removal rate could be increased 40 times compared with the traditional chemical mechanical polishing. Liu et al. [15,16,17] used a cold plasma jet to assist diamond tools in cutting ferrous metals. Cold plasma-assisted machining could reduce the graphitization temperature of diamond tools and inhibit tool wear. However, the behaviors and mechanism of the micromechanics on the ferrous metal surface under the action of plasma need to be explored further.

In order to reveal the change of material properties with APPT, and it can provide a theoretical basis to expand the application of atmospheric pressure plasma technology in the precision machining of pure iron materials. In this work, the surface wettability, tensile mechanical and micromechanical behaviors of pure iron with and without APPT modification were studied. The mechanism of the change in behavior was studied using the first-principles and molecular-dynamics methods. The surface properties and modification mechanism of pure iron modified by atmospheric pressure plasma were analyzed.

## 2. Materials and Methods

### 2.1. Materials

The pure iron material used in this paper is the original bar produced by Taiyuan Iron and Steel Co., Ltd. in China, and the brand is DT4E. The pure iron material was cold forged into a sample under normal temperature. After being treated at 700 °C for 2 h, and it was cooled in the furnace. The grain size of the pure iron was approximately grade six.

### 2.2. Application of the Atmospheric Pressure Plasma Treatment (APPT) Method

**Figure 1:**
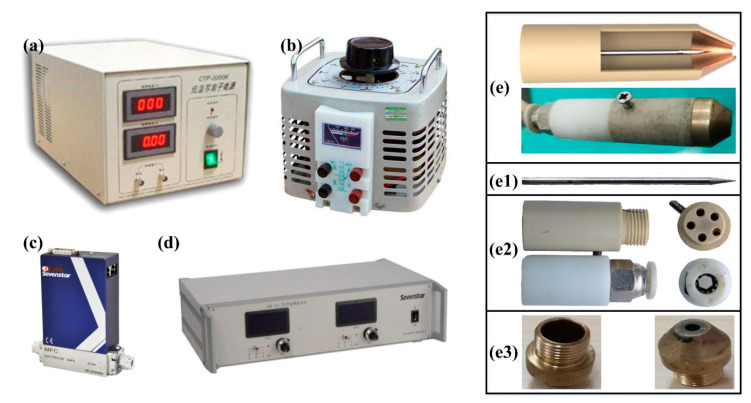
Components of atmospheric pressure cold plasma generating system: (**a**) alternating current (AC) power of low frequency, (**b**) voltage regulator, (**c**) gas mass flow controller, (**d**) gas flow meter, (**e**) plasma generator, (**e1**) tungsten needle, (**e2**) insulated cavity, (**e3**) copper electrode.

As shown in Figure 1, the APPT generating system includes an alternating current (AC) power supply (CTP-2000K, Suman, Nanjing, China), voltage regulator (TDGC2, Zhengtai, Hangzhou, China), gas mass flow controller (CS200, Sevenstar, Beijing, China), gas flow meter (D08-2F, Sevenstar, Beijing, China) and plasma generator. The details of plasma generator with bare electrode discharge are shown in Figure 1(e,e1–e3). Atmospheric pressure plasma discharges as bare electrode discharge, and the space of the electrodes is filled with high-purity nitrogen (99.99%), which can produce a stable atmospheric pressure plasma jet at a low voltage. During the test, the plasma power supply frequency was set to 58.6 kHz, the driving voltage was 1.53 kV, the nitrogen pressure was 0.5 MPa, the nitrogen flow rate was 12 L/min and the nozzle diameter was 2.5 mm. The stable plasma jet with a length of 16 mm was generated after the working gas flows through the insulated cavity and discharged under the given parameters.

**Figure 2:**
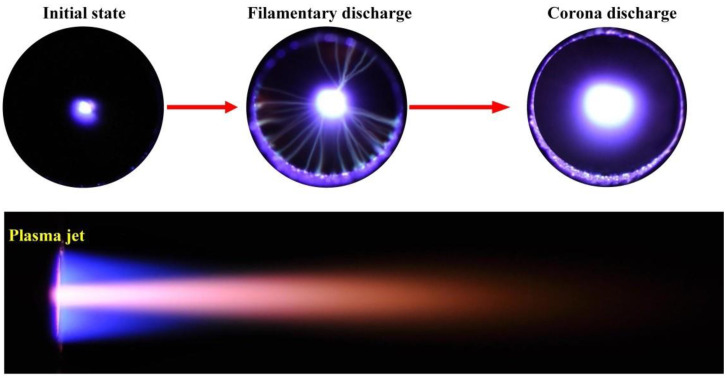
Discharge process and stable plasma jet under *U* = 1.53 kV.

**Figure 3:**
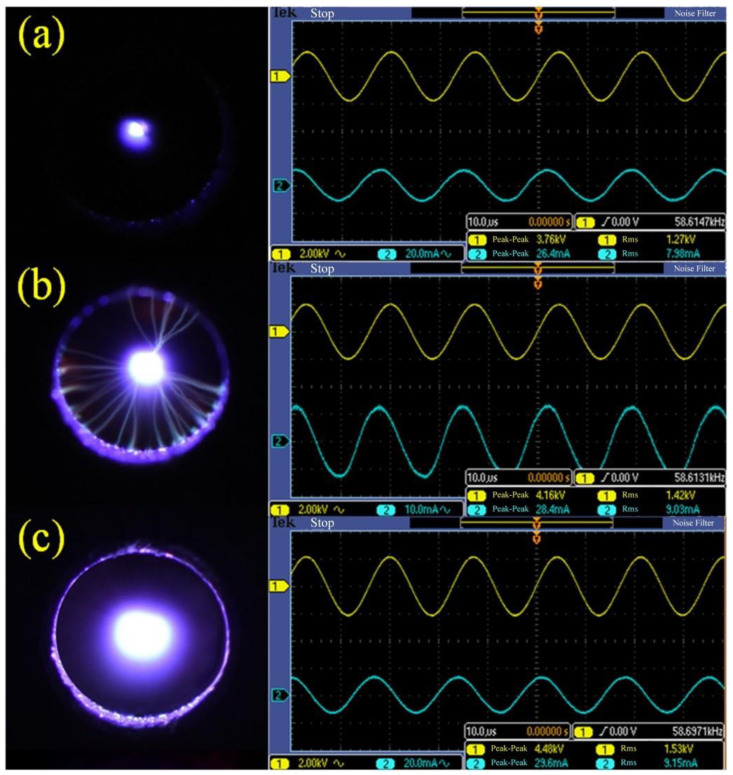
Oscillogram of plasma under different discharge voltages: (**a**) *U* = 1.27 kV, (**b**) *U* = 1.42kV, (**c**) *U* = 1.53 kV.

As shown in Figure 2, the discharge state was changed with the increasing voltage. The stable and long plasma jet was not generated until the discharge voltage *U* = 1.53 kV, and the corona discharge was found between the two electrodes at the same time. In order to explore the relationship between the form of discharge and the increasing voltage, oscillograms of plasma under different discharge voltage were tested by oscilloscope (DPO 2014B, Tektronix, OH, USA), which was shown in Figure 3. The filamentary discharge was found when the voltage increased from 0 V to 1.27 kV (Figure 3a). However, the generated plasma jet was too short to spray from the insulated cavity at the same time. When the discharge voltage increased to 1.42 kV, filament discharge occurred between the high-voltage tungsten needle electrode and the ground electrode (Figure 3b), the generated plasma jet of which was unstable. When the discharge voltage increased to 1.53 kV, as shown in Figure 3c, the discharge state changed from filamentary discharge to corona discharge, and a large-scale, uniform and stable plasma jet could be formed at this time.

### 2.3. Characterization of Material Surface Properties

The machined 50 mm × 50 mm × 6 mm samples were used in wettability tests. The workpiece surface was divided into six 10 mm × 10 mm rectangular squares. The rectangular squares were treated for different times (2 s, 4 s, 6 s, 8 s, 10 s, 12 s, 14 s, 16 s, 18 s, 20 s and 22 s) by N plasma. The red water droplets were dropped onto the surface of the processed workpiece. The contact angle was measured by an optical contact-angle meter (SL200KS, KINO, Boston, MA, USA). The volume of water droplets used in the measurement was 5 μL.

The 15 mm × 15 mm × 8 mm samples were used in the nanoindentation tests. After lapping and polishing, the surface roughness of the samples was controlled to within 10 nm. A Nano Indenter XP (MTS, Eden Prairie, MN, USA)-type full-automatic nanomechanical test system was used for the nano-indentation tests. The tests were carried out at room temperature with a Berkovich indenter with a nominal front-end radius of 2 μm, and an angle α between the center line and surface of 65.3°. The linear loading mode was used to drive the indentation tester, and the maximum indentation loads were 100 mN, 300 mN, 400 mN and 500 mN. The loading, holding and unloading times were 30 s, 10 s and 30 s, respectively. To ensure the accuracy of the experimental results, the experiments were repeated three times for each set of test parameters, and the distance between every two indentations was larger than 100 μm. According to the above loading parameters, nano-indentation tests were carried out on the polishing surface of the workpiece that had been pretreated by atmospheric pressure plasma for 30 s, and then the load-displacement curve and indentation hardness were compared.

**Figure 4:**
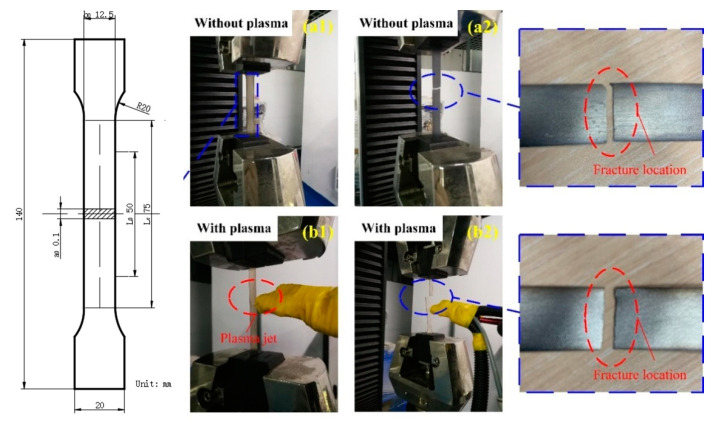
Sketch map of tensile specimens and tensile test.

The tensile mechanical properties of the materials without and with APPT modification were tested on a material tensile testing machine (CTM8050, Shenzhen, China). The measurement method refers to Chinese national standard GB/T 228.1-2010 [18]. Non-proportional specimens with a rectangular cross section were used in the pure iron tensile test, with the following sizes: original width *b*_0_ = 12.5 mm, original gauge distance *L*_0_ = 50 mm, parallel length *L_c_* = 75 mm and original thickness *a*_0_ = 0.1. A sketch of the tensile specimens and tensile tests is shown in Figure 4. The constant loading rate was set to 0.1 mm/s. When the preload reaches 1 N, the data were collected until the sample broke.

### 2.4. Simulation Method

The surface physical and chemical process of the nitrogen plasma that was induced by APPT treatment on the pure iron was studied by molecular dynamics simulations and the first principles method. Molecular dynamics simulations were used to study the evolution of atomic structure on the surface of pure iron during treatment, and the first principles method was used to study the bonding between the nitrogen and iron atoms. The first principles method was described below. The Perdew–Burke–Ernzerhof (PBE) exchange-correlation functional and the projector-augmented-wave (PAW) approach were used. The K-points setting were 10 × 10 × 1. The plane-waves cutoff energy was 400 eV. A total of 50 atoms were set in the system, including 48 Fe atoms and 2 N atoms. The thickness of the vacuum layer was set to 15 nm. Then the first principle calculation was based on Vienna ab initio simulation package (VASP). All strain rates were less than 1%. Due to the limitation of computational resources, a SCAITools (2.92, Synopsys, CA, USA) model was set up with a small calculation system and small grain size. Therefore, most of the system was occupied by the grain boundary of amorphous structure. If a larger system was built, the grain size will be increased, and the proportion of grain boundaries will be reduced, and then it will look more like polycrystalline. The energy of the systems was minimized before the tensile deformation and nano-indentation. In this paper, uniform tensile deformation speed and nano-indentation speed were used. The energy of the systems was minimized before the tensile deformation and nano-indentation. When setting the simulation parameters, it is necessary to set the correct molecular dynamics parameters from the literature [19,20,21], namely, a plasma energy range of 0.1 eV–100 keV. According to a previous study [17], the plasma energy was set to 10 eV. The microcanonical ensemble was used in the simulated plasma treatment process, and the modified embedded-atom method potential was used as the potential function [22,23]. A Langevin (12Dec18, Sandia, NM, USA) hot bath was used to relax the system before treatment. Large-scale Atomic/Molecular Massively Parallel Simulator software (LAMMPS, 12Dec18, Sandia, NM, USA) was used for the molecular dynamics and the Open Visualization Tool (OVITO, 3.1.3, Technical University of Darmstadt, Germany) software was used for visualization.

## 3. Results and Discussion

### 3.1. Surface Wetting Behavior of Pure Iron

**Figure 5:**
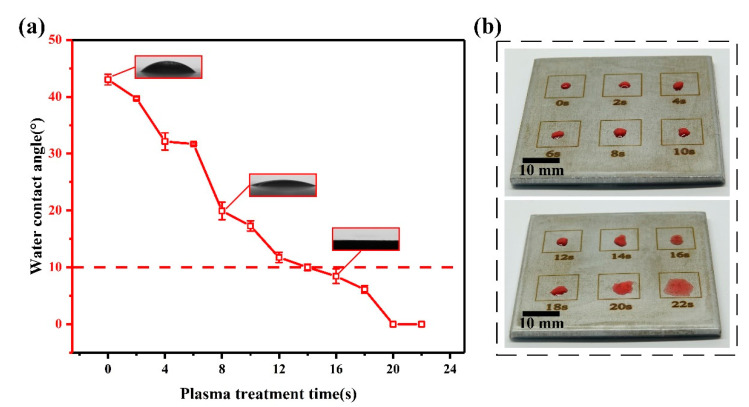
Test results of surface wettability of pure iron with atmospheric pressure plasma treatment (APPT) treatment: (**a**) change rule of water contact angle at plasma treatment time, (**b**) state of liquid drop on pure iron surface.

The results from the surface wettability test on pure iron with and without APPT treatment are shown in Figure 5. Figure 5a shows that without APPT treatment, the contact angle of the pure iron surface was 43°. After treatment by APPT for 16 s, the contact angle of the pure iron surface changed to 8°, which indicates that the modified surface is superhydrophilic. As shown in Figure 5b, an increase in plasma treatment time results in a gradual increase in surface hydrophilicity of the pure iron, which indicates that the cooling lubricating medium enters the tool-workpiece contact area easily during cutting. Therefore, it is possible to reduce the friction and cutting heat during APPT-assisted cutting.

The main reasons for the improved material wettability by APPT are based on following aspects [24,25,26,27]. During the atmospheric pressure plasma discharge, the molecular ground state N_2_ (X1$\sum_{g}^{+}$) are excited by some electrons (less than 10 eV) to form a large number of metastable nitrogen molecules N_2_ (A3$\sum_{u}^{+}$)and oxygen-containing active particles such as NO, O and OH. Active particles contained in plasma such as electrons and metastable nitrogen molecules (about 6.2 eV) can interrupt most organic chemical bonds, such as C-C bonds (about 3.45 eV) and C-O bond (about 3.48 eV) [17]. The atmospheric pressure plasma jet introduces hydrophilic groups onto the material surface. The hydrophilic groups that are attached to the material surface can improve the material surface energy and enhance the hydrophilicity. In Section 3.4, we explain the reason for this behavior through molecular dynamics and first principles.

### 3.2. Macromechanical Behavior of Pure Iron

**Figure 6:**
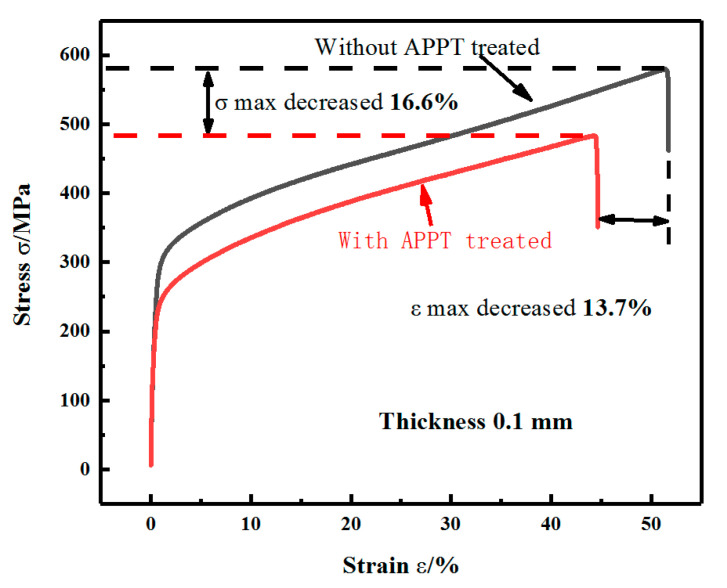
Stress-strain curve of sample with and without APPT.

Figure 6 shows the stress-strain curve of a pure iron tensile specimen with and without APPT treatment. As shown in Figure 6, the maximum tensile stress σ_max_ of the original specimen was 579.1 MPa, whereas that of the APPT-treated specimen was 483.3 MPa. The maximum tensile strain rate ε_max_ of the original and treated specimens is 51.7% and 44.6%, respectively. After plasma treatment, the maximum tensile stress and tensile strain of the sample decreased by ~16.6% and 13.7%, respectively. The tensile test results show that the yield strength and fracture stress of pure iron can be decreased by APPT treatment.

### 3.3. Surface Micromechanical Behavior of Pure Iron

**Figure 7:**
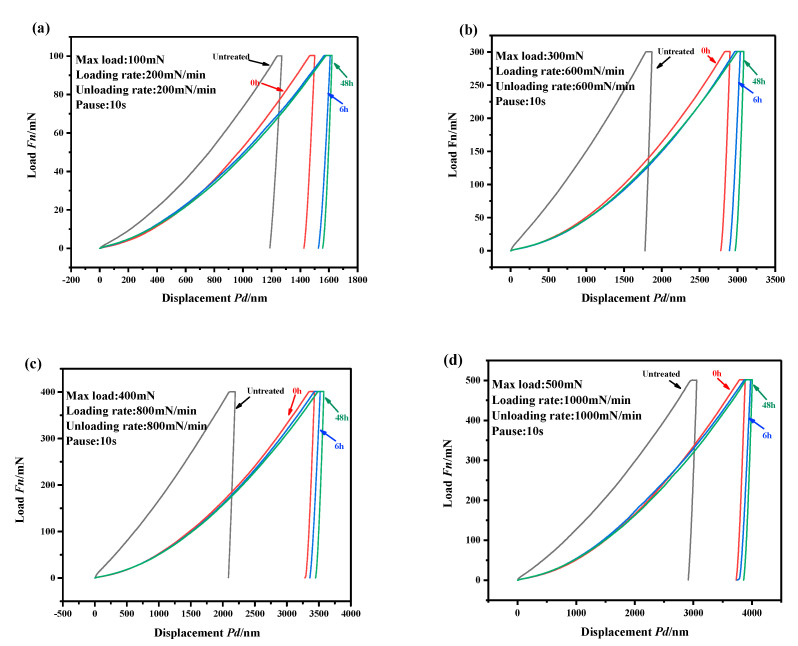
Force-displacement curves for different loads: (**a**) maximum indentation load is 100 mN, (**b**) maximum indentation load is 300 mN, (**c**) maximum indentation load is 400 mN, (**d**) maximum indentation load is 500 mN.

The surface micromechanical behaviors of pure iron with and without APPT were characterized by using a nanoindentation instrument. The force-displacement curves under various loads are shown in Figure 7. Figure 7 shows that when the maximum indentation loads were set to 100–500 mN, the maximum indentation depth *h_max_* and residual indentation depth *h_f_* of the plasma-treated samples increased significantly (~50%) compared with those of the untreated samples. An increase of storage time (0–48 h) resulted in a slow increase in varying degree of *h_max_* and *h_f_*.

**Figure 8:**
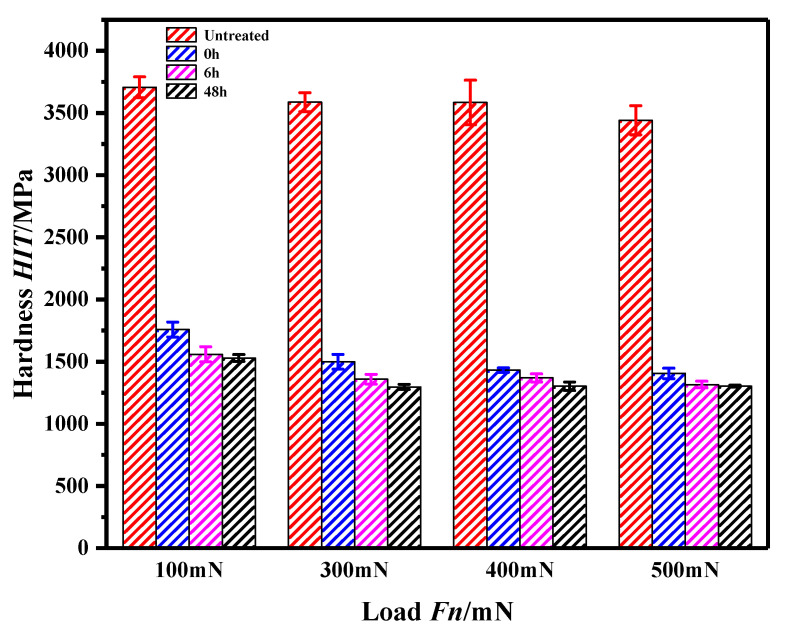
Curves of surface microhardness of pure iron under different loads and treatments.

The microhardness curves of the pure iron surface under different conditions are shown in Figure 8. Figure 8 shows that for the same pressing load, the microhardness of the pure iron surface without APPT ranged from 3600–3700 MPa, and that of the pure iron surface with APPT decreased by ~60%. When the pure iron surface was treated by APPT, the microhardness of the pure iron surface decreased slightly with placing time for 0 h, 6 h and 48 h. This phenomenon shows that the APPT can reduce the surface hardness of pure iron materials and soften the pure iron material surface.

### 3.4. Molecular Dynamics Simulation Results

Molecular dynamics and first-principles calculations were used to simulate the physicochemical process of the interaction between the plasma and the pure iron surface, and thus the influence mechanism of plasma treatment on the micromechanical properties and hydrophilicity of pure iron was proposed.

#### 3.4.1. Physical and Chemical Process of Pure Iron Treated by APPT

**Figure 9:**
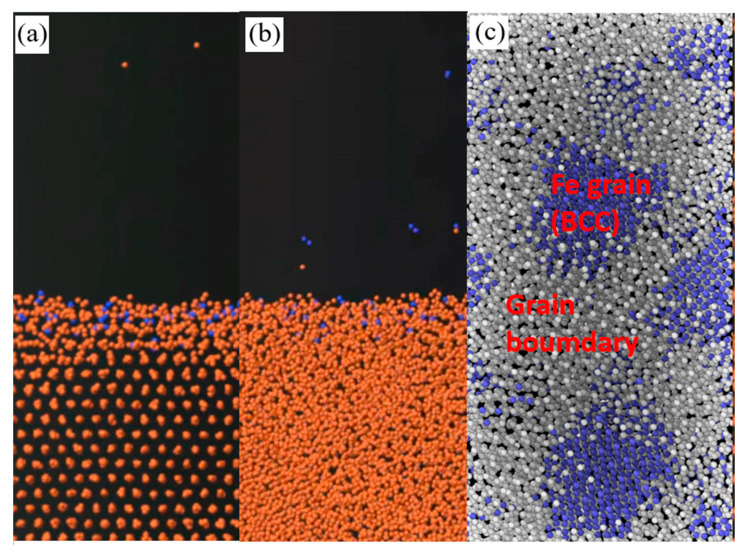
Stacking structure of Fe atoms treated with N plasma: (**a**) single-crystal Fe treated at 10 eV, (**b**) polycrystalline Fe treated at 10 eV, (**c**) grain boundary of polycrystalline Fe treated at 10 eV.

**Figure 10:**
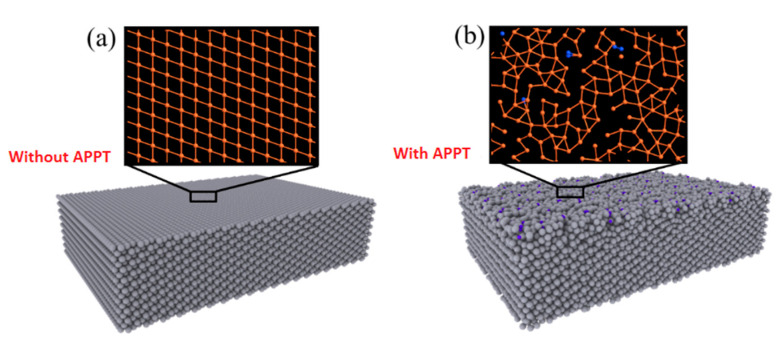
Stacking structure of Fe atoms: (**a**) without and (**b**) with APPT.

The stacking structure of Fe atoms that were treated by a N plasma with 10 eV energy is shown in Figure 9. Because the energy of the atmospheric pressure plasma is insufficient for material removal, the physical and chemical reactions between the 10 eV plasma and its impact on the surface were chosen for analysis. The stacking structure of Fe atoms treated with N plasma is shown in Figure 9. The atomic stacking structure of polycrystalline Fe and single-crystal Fe that were treated with a 10 eV plasma treatment was very similar (the atomic structure of polycrystalline Fe showed obvious grains and grain boundaries and was not amorphous), as shown in Figure 7a–c. Some Fe atoms were separated from the pure iron surface after irradiation by the 10 eV plasma, and Fe atom vacancy defects were formed. Some N atoms remained in the Fe lattice, which formed the atomic stacking structure as shown in Figure 10b.

**Figure 11:**
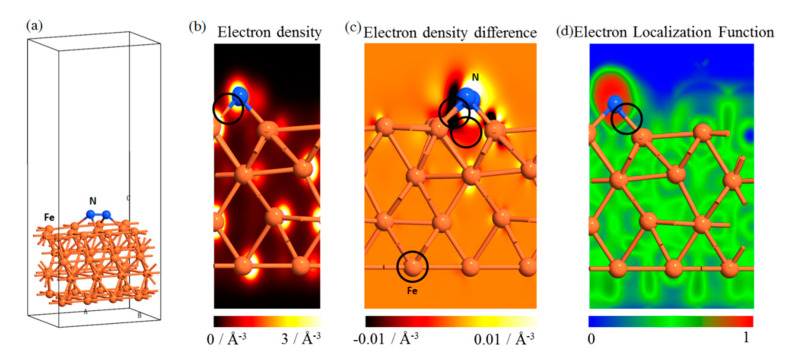
Electronic structure of Fe–N system by first principles: (**a**) model used in the first-principles calculation, (**b**) electron density distribution of Fe–N system, (**c**) differential charge density of Fe–N system, (**d**) electron localization function of Fe–N system.

To study the chemical bonding and properties between the N and Fe atoms on the iron surface, the electronic structure of the Fe-N system was calculated by first principles, as shown in Figure 11. The charge density between the N and Fe atoms is low (Figure 11b), which indicates that the electron orbit coupling between the N and Fe atoms is relatively weak. Therefore, the possibility of covalent bond formation is relatively low. Strong charge transfer occurs between the Fe and N atoms (Figure 11c) and the possibility of ion bond formation is high because of the electron transfer from Fe to N atoms. The electron localization function shows that the electron is localized strongly near the N and Fe atoms, which is consistent with the ion bond characteristics, as shown in Figure 11d. The above analysis indicates that the combination of Fe and N atoms occurs mainly in the form of ionic bonds [22]. Figure 11c shows that the presence of N atom reduces the electron density between Fe–Fe atoms. Fe–Fe atoms are combined by metal bonds. This requires electron participation and is equivalent to a weakening of the metal bond strength between Fe atoms. However, this weakening effect is localized. The charge density of the Fe atom that is closer to the N atom will be affected, whereas the charge density of the Fe atom farther from the N atom will not be affected. Therefore, the effective depth of plasma treatment depends mainly on the injection depth of the N atom.

#### 3.4.2. Micromechanical Properties of Pure Iron Treated by APPT

**Figure 12:**
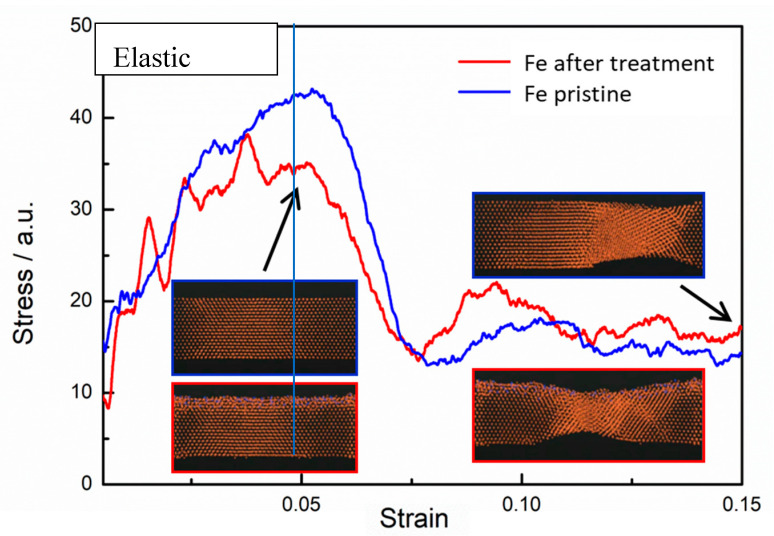
Uniaxial tensile stress-strain curves of iron with and without N plasma treatment.

**Figure 13:**
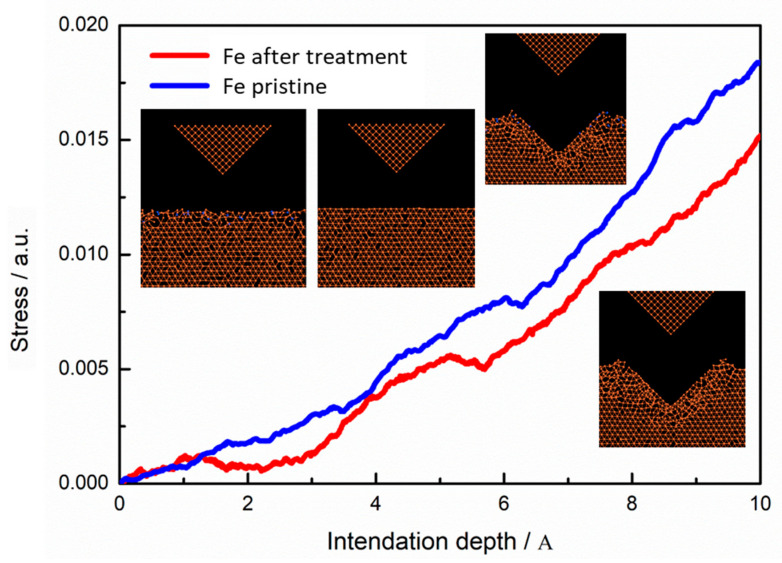
Simulation results of nano-indentation.

By using the atomic stacking structure model of a plasma-treated iron surface (as shown in Figure 10), the uniaxial tensile stress-strain curves of iron with and without N plasma treatment were obtained by molecular dynamics methods, as shown in Figure 12. The yield stress of the iron decreased after plasma treatment, which is consistent with the tensile experiment results (as shown in Figure 4). According to the evolution of atomic structure in the uniaxial tensile process given by molecular dynamics, the reason for the decreasing yield stress was analyzed. In uniaxial tension, the elastic deformation of iron occurred first, which is represented by Fe–Fe bond tension. Then the Fe lattice Fe was distorted, and plastic deformation of the iron occurred. A maximum stress appeared in the elastic deformation stage, and the magnitude of the stress depended on the number and strength of Fe–Fe bonds. The results in Figure 10 show that many Fe–Fe bonds are broken, and the existence of N atoms weakened the strength of the Fe–Fe bonds. After plasma treatment, there was a clear amorphization on the surface (shown in Figure 9, Figure 10, Figure 12 and Figure 13). Therefore, surface amorphization may be another important reason for the reduction of yield stress [28,29,30]. Therefore, the plasma treatment decreased the yield stress of iron.

The change of iron microhardness with and without N plasma treatment was studied. The stress-depth curves of nano-indentation from molecular dynamics are shown in Figure 13. An increasing number of atomic iron structures yielded and plastic flow occurred during constant indenter pressing. The material microhardness is determined by the yield stress. The microhardness will be reduced correspondingly because of a reduction in iron yield stress by plasma treatment. The simulation results are consistent with the experimental results in Section 3.3 (as shown in Figure 8).

#### 3.4.3. Wetting Behavior of Pure Iron Treated by APPT

**Figure 14:**
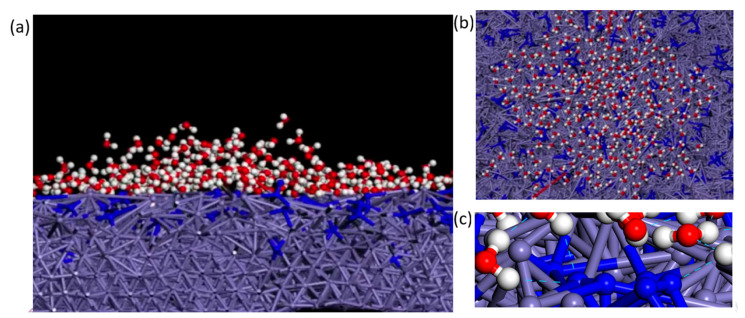
Wetting behavior simulation results: (**a,b**) superhydrophilicity of iron surface with plasma treatment, (**c**) hydrogen bond between N atom and water molecule.

Molecular dynamics was used to simulate the iron surface hydrophilicity with N plasma treatment. The simulation results are shown in Figure 14. A constant temperature (NVT) ensemble was used in the simulation, the temperature was set to 298 K and the Condensed-phase Optimized Molecular Potentials for Atomistic Simulation Studies (COMPASS II) force field in the material studio was used [31].

Figure 14 shows that water molecules can spread on the iron surface, which indicates that the treated surface has a high hydrophilicity, which is consistent with the superhydrophilicity observed in the experiment. The formation of a superhydrophilic surface may be related to residual N atoms. N atoms can form hydrogen bonds with water molecules [23,32], and the hydrogen bond energy is higher than that of the Van der Waals’ forces. Therefore, N atoms can improve the binding energy between water and the iron surface, which enhances the iron surface hydrophilicity. This result is consistent with the experimental results in Section 3.1 (as shown in Figure 3). The atom model color represents different atoms. Red represents the O atom, white represents the H atom, dark blue represents the N atom, and lavender represents the Fe atom.

In conclusion, polar ion adsorption on the crystal surface can induce a variety of physical and chemical processes and change the mechanical and physical properties of the crystal surface materials. If N plasma is used to assist with the cutting of pure iron materials, active particles in the plasma will interact with the cutting area via complex thermodynamic reactions, which will reduce the microhardness, fracture strength and plastic deformation resistance of the processed pure iron materials, and facilitate cutting. The hydrophilic surface of the material that was modified by APPT was conducive to sufficient cooling and lubrication of the cutting fluid, and the reduction in surface yield stress and hardness of the material that was modified by the plasma reduced the cutting force. The research results provide a theoretical basis for application of APPT in the ultraprecision-assisted cutting of pure iron materials [17]. Therefore, the technology of APPT-assisted cutting to process pure iron materials is expected to yield an excellent processing performance.

## 4. Conclusions

Atmospheric pressure plasma can improve the surface wettability of pure iron. The modified surface is superhydrophilic. The yield strength and fracture stress of pure iron was reduced by APPT. After plasma pretreatment, the surface indentation hardness decreased by ~60%. When N plasma impacts the surface of pure iron, Fe atoms escape from the surface and Fe vacancy defects form. The metal bonds between the surrounding Fe atoms are weakened. Ion impact fractures the Fe–Fe bonds, and N atoms reduce the Fe–Fe bond strength, which leads to a decrease in yield stress and microhardness. After plasma treatment, N atoms on the material surface form hydrogen bonds with the water molecules. This increases the binding energy between the water and the material surface and improves the material hydrophilicity.
